# Probiotic Encapsulation: Bead Design Improves Bacterial Performance during In Vitro Digestion (Part 2: Operational Conditions of Vibrational Technology)

**DOI:** 10.3390/polym16172492

**Published:** 2024-08-31

**Authors:** Yesica Vanesa Rojas-Muñoz, María de Jesús Perea-Flores, María Ximena Quintanilla-Carvajal

**Affiliations:** 1 Universidad de La Sabana, Facultad de Ingeniería, Maestría en Diseño y Gestión de Procesos, Campus Universitario del Puente del Común, Chía 250001, Cundinamarca, Colombia; yesicaromu@unisabana.edu.co; 2 Instituto Politécnico Nacional, Centro de Nanociencias y Micro y Nanotecnologías, Unidad Profesional “Adolfo López Mateos”, Luis Enrique Erro s/n, Zacatenco, CDMX C.P. 07738, Mexico; mpereaf@ipn.mx; 3 Universidad de La Sabana, Facultad de Ingeniería, Grupo de Investigación de Procesos Agroindustriales (GIPA), Campus Universitario del Puente del Común, Chía 250001, Cundinamarca, Colombia

**Keywords:** probiotic, encapsulation, vibration technology, functional food, INFOGEST

## Abstract

The development of functional foods is a viable alternative for the prevention of numerous diseases. However, the food industry faces significant challenges in producing functional foods based on probiotics due to their high sensitivity to various processing and gastrointestinal tract conditions. This study aimed to evaluate the effect of the operational conditions during the extrusion encapsulation process using vibrating technology on the viability of *Lactobacillus fermentum* K73, a lactic acid bacterium with hypocholesterolemia probiotic potential. An optimal experimental design approach was employed to produce sweet whey–sodium alginate (SW-SA) beads with high bacterial content and good morphological characteristics. In this study, the effects of frequency, voltage, and pumping rate were optimized for a 300 μm nozzle. The microspheres were characterized using RAMAN spectroscopy, scanning electron microscopy, and confocal laser scanning microscopy. The optimal conditions for bead production were found: 70 Hz, 250 V, and 20 mL/min with a final cell count of 8.43 Log_10_ (CFU/mL). The mean particle diameter was 620 ± 5.3 µm, and the experimental encapsulation yield was 94.3 ± 0.8%. The INFOGEST model was used to evaluate the survival of probiotic beads under gastrointestinal tract conditions. Upon exposure to in vitro conditions of oral, gastric, and intestinal phases, the encapsulated viability of *L. fermentum* was 7.6 Log_10_ (CFU/mL) using the optimal encapsulation parameters, which significantly improved the survival of probiotic bacteria during both the encapsulation process and under gastrointestinal conditions compared to free cells.

## 1. Introduction

Heart disease and stroke are among the most common diseases, and high cholesterol levels increase the risk of suffering from them. According to the World Health Organization (WHO), it is estimated that high cholesterol causes 2.6 million deaths annually. *Lactobacillus fermentum* K73 is a lactic acid bacterium (LAB) isolated from fermented food on the Colombian Atlantic coast. According to the in vitro studies conducted, it has potential as a hypocholesterolemic probiotic [1]. Probiotic cells contribute to the intestinal microbial balance; the intestine is the ideal site of action for their release in the indicated dose. They must overcome the conditions of the gastrointestinal (GI) tract, a hostile environment due to the presence of bile acids, antimicrobial compounds, and degrading enzymes [2,3,4]. Previous studies have demonstrated that the microencapsulation technique can protect probiotic cells against adverse gastrointestinal conditions [5,6,7,8]. Microencapsulation consists of the formation of carriers called microcapsules, microspheres, or microparticles, depending on the distribution of the active ingredient in the matrix. These carriers transport one or more immobilized bioactive compounds, which can be enzymes, antioxidants, flavorings, vitamins, oils, or cells [9,10]. Different microencapsulation techniques such as extrusion, emulsion, coacervation, liposome entrapment, spray-drying, and fluid bead coating have been developed. Among the different probiotic encapsulation technologies, the extrusion technique is widely used to encapsulate bacterial cells in the food industry due to the benefits it brings: it does not use high temperatures or inorganic solvents, it is low-cost, and the use of food-grade bioavailable materials ensures high viability of the encapsulated cells [11,12,13].

Extrusion consists of mixing the probiotic with the encapsulating material, then pumping the mixture through an injector system into a hardening solution that immediately forms droplets or microcapsules by the ionotropic gelation physicochemical process [14,15,16]. According to [17], if the droplet formation is controlled during the encapsulation process by vibration frequency, the technique is named *prilling* or the extrusion with vibrational technique. The characteristics of the droplets are diverse depending on the selection of the encapsulator’s operating conditions, i.e., nozzle diameter, flow rate of the laminar jet, vibration frequency, and viscosity of the hydrocolloid solution [17,18]. 

The BUCHI 390 Pro Encapsulator is equipped with a stroboscopic lamp, which is important as it provides useful parameters for the formation of the particles by visualizing bead formation in real time. Once the parameters are reached, a chain of droplets appears. However, there is a functional operating range of these parameters, which will determine the size and shape of the resulting bead. Some studies have reported that larger capsules provide more protection to the encapsulated material [19]. Consequently, there is a need to optimize the operating conditions of the equipment in order to provide maximum protection for the encapsulated bioactives.

The objective of this work was to investigate the effect of the operating parameters of the vibratory extrusion encapsulation process on the viability of *Lactobacillus fermentum* K73. This study is a continuation of our previous work [20], in which the optimal mixture of wall materials for the beads was determined. A Response Surface Methodology (RSM) was applied to optimize the production process of probiotic microcapsules; the goal was to have the highest viability and microencapsulation efficiency. After production of the encapsulates under optimal conditions, their resistance to simulated conditions of the gastrointestinal tract was determined following the INFOGEST protocol. Finally, the beads were characterized using various microscopy technologies.

## 2. Materials and Methods

### 2.1. Materials

The reagents used for the growth and preservation of the bacteria were sweet milk whey (crude protein 11%, crude fat 1.5%, lactose 61%) (Saputo Ingredients, Lincolnshire, IL, USA), yeast extract (Scharlau Microbiology, Barcelona, Spain), MRS agar and broth (Man Rogosa and Sharpe), and peptone water and glycerol (PanReac AppliChem, Barcelona, Spain). The encapsulation process used sodium alginate (M/G 0.9; MW 1.40 × 104 g/mol) (Sigma-Aldrich (Saint Louis, MI, USA), calcium chloride dihydrate (PanReac AppliChem, Barcelona, Spain), and sodium citrate. All enzymes used in digestion tests—pancreatin P7545, lipase L3126, and ox-bile extract 70,168—as well as the fluorochromes, were acquired from Sigma-Aldrich (Saint Louis, MI, USA).

### 2.2. Methods

#### 2.2.1. Strain and Biomass Production

The *L. fermentum* K73 strain used in this study is preserved in the GenBank Usab-Bio of the Department of Engineering, Universidad de La Sabana (Colombia). The stock cultures were stored at −80 °C (Ultra-freezer, Precisa, Hangzhou, China) in 2 Ml vials containing bacterial culture grown (12 h, 37 °C) in MRS broth and glycerol (40% *v*/*v*) as a cryoprotective agent in a volume ratio of 1:1. The culture medium was prepared according to [21]; briefly, the bioreactor (1.3 L BioFlo 110; New Brunswick Scientific Co., Enfield, CT, USA) working load was 800 mL (sweet whey 8% *w*/*v*, yeast extract 0.22% *w*/*v*), and it was adjusted to a final pH of 5.5 with 1 M HCl. Operational conditions were set to a temperature of 37 °C and agitation speed of 100 rpm for 10 h. After sterilizing the culture medium at 121 °C for 15 min., *L. fermentum* K73 was inoculated at 10% (*v*/*v*).

#### 2.2.2. Encapsulation Process

##### Preparing Solutions

Sodium alginate 1.5% (*w*/*w*) solution was prepared with distilled water at 90 °C for 30 min under magnetic stirring (250 rpm) [22]. Calcium chloride (CaCl_2_ · 2H_2_O) hardener solution (100 mM) was prepared with deionized water and adjusted to a final pH of 4.5. All solutions were sterilized.

##### Probiotic Encapsulation

The encapsulation process consisted of the preparation of the optimal mixture determined by Rojas-Muñoz et al. [20]. In summary, the mixture of the wall materials was prepared as follows: sodium alginate at 0.61% and sweet whey at 0.39%, stirring gently until homogenized. It is important to highlight that the culture medium based on sweet whey functions as one of the constituent materials in the mixture of the pearl wall materials. The mixture was pumped through a 300 μm nozzle into the encapsulator (B-395 Pro Encapsulator, BUCHI, Flawil, Switzerland). The resulting droplets were collected in the hardener solution at a height of 9 cm. The volume ratio of the mixture to the hardener bath was 1:4. The beads formed immediately were kept in agitation at 80 rpm for 20 min to promote efficient gelation of the particles [23]. For viability analyses, fresh beads were used, while for SEM analysis, the particles were freeze-dried (Labconco, USA) at −40 °C for 24 h.

The optimization study of the encapsulation conditions was conducted using an experimental design affecting the following factors:

Vibration frequency (Factor A): This factor controls the rate at which the nozzle vibrates, which is crucial for breaking up of liquid jets into small *prills* (droplets). The frequency range evaluated was from 70 to 4000 Hz.

Electrode tension (Factor B): This is the electrical potential applied between the nozzle and an electrode. It is used to prevent coalescence of the droplets during jet break-up and/or when entering the gelling bath. The voltage range evaluated was from 250 to 2500 V.

Syringe pump rate (Factor C): This describes the rate at which the syringe pump delivers the mixture into the encapsulator. This rate affects the flow rate and consistency of the droplets formed. The pump rate range evaluated was from 6 to 20 mL/min.

The optimal mixture with a viscosity of 151.1 mPa·s was fed into the encapsulator according to the run evaluated (see Table 1). The response variables to be evaluated were as follows: bacterial viability Log_10_ (CFU/mL) after the encapsulation process and percentage of encapsulation efficiency. The design included 20 runs with 5 replicates. The specialized software Design Expert (v11.0.0, Stat-Ease Inc., Minneapolis, MN, USA) was used for the analysis of variance (ANOVA) and generation of contour plots.

The optimization criterion was desirability (D ≥ 0.7). This involved the selection of conditions that maximized the viability and encapsulation efficiency of *L. fermentum* K73. The viability of the encapsulated microorganism with optimal conditions was evaluated. The experimental response was statistically compared with that predicted by the model.

(1) $$\%\;Error=\frac{Predicted\;variable-Observed\;variable}{Predicted\;variable}\cdot 100$$

##### Cell Release

The release of the probiotic cells from the bead matrix was achieved using the technique documented by [24]. Briefly, 5 g of beads were diluted 1:10 (*v*/*v*) in a sodium citrate solution of 5% (*w*/*v*) and subjected to homogenization in a vortex (VG 3, IKA, Werke, Germany) for 60 s. Subsequently, cell viability was determined using the plate count method.

##### Cell Viability

The cell count of *L. fermentum* K73 for all the proposed experiments was carried out by making 1:10 (*v*/*v*) serial dilutions in 0.1% (*w*/*v*) peptone water. Plating was performed on MRS agar from dilution 1 to 7 in triplicate. Incubation conditions were 37 °C for 24–48 h under aerobic conditions. Plates containing up to 250 colonies were counted. The result is reported as the logarithm of the final colony-forming units per milliliter (CFU/mL).

##### Encapsulation Efficiency (EE)

Cell entrapment efficiency was calculated as the difference between viable cell count before and after the microencapsulation process [25,26].

(2) $$EE\;\%\;=(log{\left(CFU/mL\right)}_{N_{2}}/log\;{\left(CFU/mL\right)}_{N_{1}})*100$$

where N_1_ and N_2_ indicate the number of viable bacteria in the mixture and released from the beads, respectively

#### 2.2.3. Beads Characterization

##### Particle Size

One hundred fresh beads were randomly selected and placed on dark paper. A digital camera (EOS 70D, Canon, Madrid, Spain) was used to photograph them. Morphometric characteristics, including diameter and circularity, were determined by processing the images with ImageJ software (V 1.50i, National Institutes of Health, Bethesda, MD, USA) [27]. The bead diameter was determined by measuring the largest distance across each bead.

##### Raman Spectroscopy

Raman spectroscopy measurements were performed following the methodology reported by [28]. A LabRAM HR800 spectrometer (Horiba Jobin Yvon, Miyanohigashi, Kyoto, Japan) coupled with an Olympus BX41 microscope was used. Raman spectra of the sample (wet bead) were recorded using a laser with emission at 784.21 nm, exposure time of 4 to 12 s, and scans from 300 to 1800 cm^−1^ at room temperature in the dark. The microscope objective used was 50 X magnification with an aperture number (AN) of 0.75. The hole opening size was 500 µm and the slit opening size was 150 µm. The spectra were processed using LabSpec V.6 software (Horiba Jobin Yvon, Longjumeau, France) and edited with Spectragryph software version 1.2.9. Spectral deconvolution was applied to remove interference from overlapping peaks using PeakFit version 4.12 (SYSTAT Software).

##### Scanning Electron Microscopy (SEM)

Some morphological parameters of the beads were observed using a scanning electron microscope (SU3500, Hitachi Co., Ltd., Matsuda, Japan) according to the protocol of [29]. The samples were placed on aluminum holders with double-sided carbon adhesive, and then coated with gold using an ionizer (SPI supplies, West Chester, PA, USA) and observed with a secondary electron detector. An accelerating voltage of 5 kV was used at different magnifications (20×–5000×).

##### Live/Dead Assay of Cells by Confocal Laser Scanning Microscopy (CLSM)

Free and encapsulated cells were stained with 5-Carboxyfluorescein Diacetate (CFDA; 1 mM) and propidium iodide (PI; 3 mM) to visualize living and dead cells simultaneously. CFDA is a cell-permeant amine-reactive that causes live cells to fluoresce green. On the other hand, PI functions as an exclusion dye that cannot penetrate living cells but readily enters dead LAB cells with damaged membranes and causes cells to fluoresce red [30]. In the assay, 100 μL of cells was mixed with 50 μL of CFDA dye and incubated at 37 °C for 20 min; then, PI dye was added in an ice bath for 15 min in the dark. Stained samples were deposited onto a coverslip and were observed by CLSM (LSM 710 NLO, Carl Zeiss, Oberkochen, Germany) using excitation wavelengths of 488 nm for CFDA and 561 nm for PI.

##### Mechanical Properties

The texture of the beads was analyzed using a texture analyzer (Brookfield CT3, Ametek, Inc., Berwyn, PA, USA) with the included software (TexturePro CV V1.6). A compression test was performed with a cylindrical probe (TA11/1000, 12.7 mm diameter). The operating conditions were a test speed of 0.5 mm/s, force calibration using a 5 kg weight, and 40% compression. Approximately 20 beads were placed in the center of the platform under the probe to perform the test. According to [31], the maximum force (N) necessary for compression represents the maximum resistance of the beads to the compression by the probe, which indirectly gives an indication of the hardness of the beads.

#### 2.2.4. Probiotic Cell Viability under INFOGEST Simulated Gastrointestinal Model

The INFOGEST protocol was used to simulate the conditions of the gastrointestinal tract proposed by [32,33]. Briefly, the oral phase was prepared to mix 5 g of the sample (beads) with 5 mL of simulated salivary fluid (SSF). The final oral phase pH was brought to 7.0 and allowed to shake (100 rpm) at 37 °C for 2 min. Then, the gastric phase consisted of adding 10 mL of simulated gastric fluid (SGF) to the oral phase and adjusting it to a final pH of 3.0 with enough volume of HCl (1 M). The sample was under agitation (100 rpm) for 2 h at 37 °C. Finally, the intestinal phase was performed by mixing the gastric phase with 20 mL simulated intestinal fluid (SIF) and the enzymes bile extract (10 mM), pancreatin (100 U/mL), and pancreatic lipase (2000 U/mL). The final pH of the intestinal phase was adjusted to 7.0, and it was incubated under the same conditions as the gastric phase. After each phase, a viability assay was carried out, and bacteria survival was determined according to Equation 3.

(3) $$Survival\;\%=(log{\left(CFU/mL\right)}_{final}/(log{\left(CFU/mL\right)}_{initial}*100$$

#### 2.2.5. Statistical Analysis

The significance test of the designs was performed by analysis of variance (ANOVA) with a confidence level of 95%. The coefficient of determination (R^2^) was used to evaluate the fit of the measurements to the regression models. Three replications were performed for all assays and the data are presented as the mean ± standard deviation. The RSM optimal design was performed using the Design-Expert software (version 11.1.0, Stat-Ease Inc., Minneapolis, MN, USA). The design included 20 runs with 5 replicates. For the optimization of the response variable, the desirability criterion of the specialized software was used.

## 3. Results and Discussion

### 3.1. Optimal Design

The selection of appropriate operating conditions for the probiotic cell encapsulation process is closely related to viability and stability. These parameters influence the size and morphology of the beads, which in turn affect the final bacterial load. In this study, an RSM optimal randomized design was evaluated. The quadratic models for the response variables viability and encapsulation efficiency were statistically significant (*p* ≤ 0.05) with non-significant lack-of-fit values (*p* ≤ 0.05). The adjusted determination coefficients (R^2^) were 0.813 and 0.802, respectively (see Table 2).

The analysis of variance (ANOVA) of the regression for the response variable viability showed that the interaction coefficient between frequency and electrode tension was statistically significant (*p* ≤ 0.05). The coefficients of the quadratic terms of electrode tension and pump rate were also statistically significant (*p* ≤ 0.05). The second-order polynomial equation in terms of all coded factors is:

(4) $$\begin{array}{c} Y\;=8.01-0.08\;A\;+0.05\;B\;+0.04\;C+0.15\;\mathrm{AB}+0.07\;\mathrm{AC}-0.37\;BC\\ -0.01\;A^{2}+0.02\;B^{2}+0.07\;C^{2} \end{array}$$

where Y is the predicted response, and A, B, and C are the coded values of the factors.

**Figure 1:**
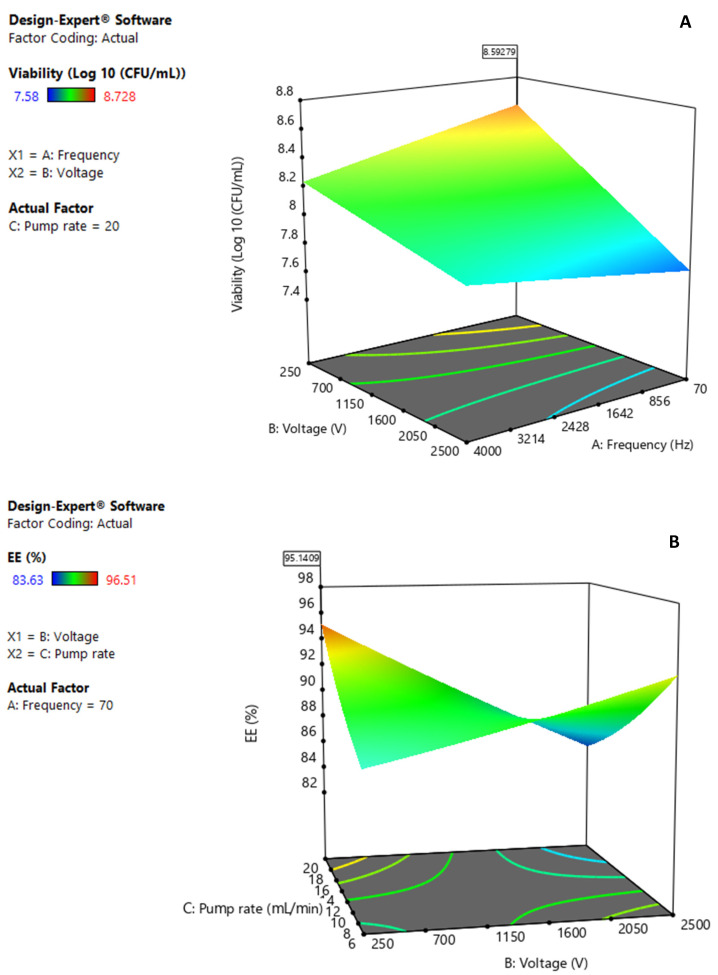
Three-dimensional surface response plots for variables: (**A**) viability (Log_10_ (CFU/mL)), (**B**) encapsulation Efficiency (%).

To determine the behavior of the main parameters on viability, the response surface plot was analyzed (Figure 1A). This diagram is a 3D graphical representation of the effect of the frequency and electrode tension on the response variable. The graph for these data shows a peak for a frequency in the interval of 70 and 120 Hz and voltage between 250 and 700 V. A similar analysis was performed for the response variable % EE, and the quadratic model in terms of all coded factors is as follows:

(5) $$\begin{array}{c} Y\;=88.66-0.98\;A\;+0.66\;B\;+0.39\;C+1.85\;\mathrm{AB}+0.59\;\mathrm{AC}-3.94\;BC\\ -0.37\;A^{2}+0.22\;B^{2}+0.73\;C^{2} \end{array}$$

Figure 1B, a 3D graphical representation of the effect of the pump rate and voltage on encapsulation efficiency, shows a relative maximum for ranges from 18 to 20 mL/min and from 250 to 700 for the C and B factors, respectively.

The models obtained from the RSM analysis regarding the performance of the microorganism *L. fermentum* K73 in the experimental design (20 total runs) allowed the selection of the optimal encapsulator parameters. The simultaneous optimization of the responses included maximizing both of the variables bacterial viability and EE. The software results of the numerical optimization for both responses were as follows: frequency (A): 70 Hz, electrode tension (B): 250 V, pump Rate (C): 20 mL/min. Beading occurs when a controlled laminar liquid jet breaks into beads of equal size if vibrated at an optimum frequency. The smaller the beads, the lower the electrostatic voltage needed to separate the chain of droplets into individual beads. The function of the electrode voltage is to prevent the beads from colliding with each other during flight and when entering the hardening solution, as the particles are equally charged and repel each other. On the other hand, flow rates are highly influenced by the viscosity of the mixture; if viscosity exceeds 100 mPa*s, a higher flow rate and a lower frequency are required to achieve a stable chain of drops, consistent with the behavior observed in the optimization of the experimental design [34,35]. This result agrees with the study of [36], who found optimal encapsulator conditions of 20 mL/min (pump rate), 250 V (voltage), and 1000 Hz (frequency) for a 450 µm diameter nozzle.

This optimal point had a desirability of 0.89. The predicted viability and EE were 8.59 Log_10_ (CFU/mL) and 95.1%, respectively. The optimal conditions were experimentally tested as a validation assay to evaluate the predictive capacity of the model. A low error rate of 1.9% was obtained with an experimental viability of 8.43 Log_10_ (CFU/mL). The encapsulation efficiency was 94.3% (error: 0.85%). The technology used for bead formation operates under low shear stress or other stress conditions, resulting in high cell survival. [37] microencapsulated *L. bulgaricus* cells in alginate–milk microspheres using vibratory technology, reporting a high similar encapsulation yield of 99%.

### 3.2. Characterization of Beads

The morphological characterization of the beads was performed immediately after encapsulation. Fresh SW-SA beads showed spherical morphologies with an average diameter of 620 ± 5.3 µm and high circularity (0.89 ± 0.2). In general, the diameter of the Ca–alginate beads are twice the diameter of the nozzle. Different studies have reported that beads loaded with probiotics have diameters between 200 and 1500 µm. However, these sizes vary widely depending on the encapsulation materials and operational conditions [12,38].

Mechanical strength should be examined as it indicates the susceptibility of the beads to different environments, transport in fluids, and exposure to shear forces, which are relevant qualities when considering the possible food applications of alginate-Ca (II) beads as probiotic encapsulating agents. The force required to compress the beads to 40% of their size was 1.22 ± 0.9 N. This result is consistent with the study carried out by [27], which evaluated the hardness of beads with an extrusion diameter of 0.35 mm and a hardening solution pH of 5.0. These results indicate that beads have adequate mechanical stability under handling which is sufficient for the consolidation of the alginate network.

**Figure 2:**
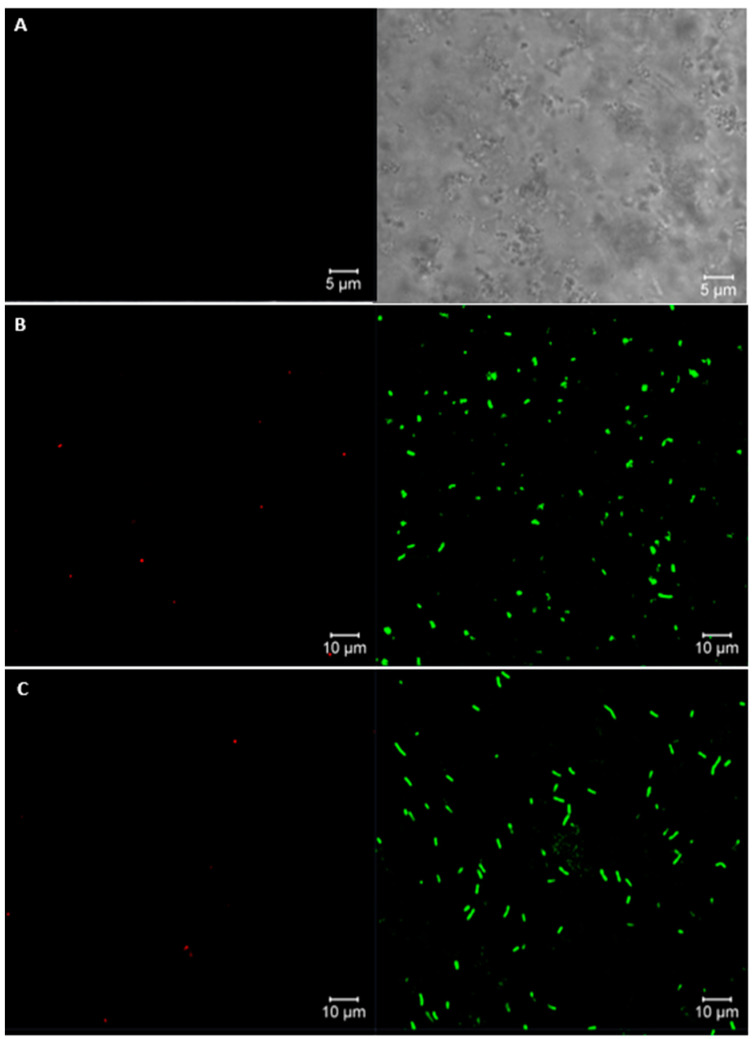
Confocal laser scanning microscopy images of bacteria viability assay: (**A**) autofluorescence control (unstained sample), (**B**) *L. fermentum K73* unencapsulated, (**C**) *L. fermentum* K73 encapsulated in SW-SA beads. Right side: live cells, left side: dead cells.

The viability of *L. fermentum* K73 was confirmed by confocal laser scanning microscopy using 5-carboxyfluorescein diacetate and propidium iodide as fluorochromes for live and dead cells, respectively. Figure 2A shows a high yield of viable bacteria in free and encapsulated bacteria, of 96.2% and 95.1%, respectively (Figure 2B,C). No apparent cell membrane damage was observed. An autofluorescence control micrograph was taken (Figure 2A). Milk protein matrices show high retention of bioactives because they represent a favorable material due to their peptic resistance and rigidity, which was confirmed through our AFM-FTIR [20] and CSLM studies. Figure S3 shows a confocal laser scanning microscopy 3D image for SW-SA bacterial beads.

**Figure 3:**
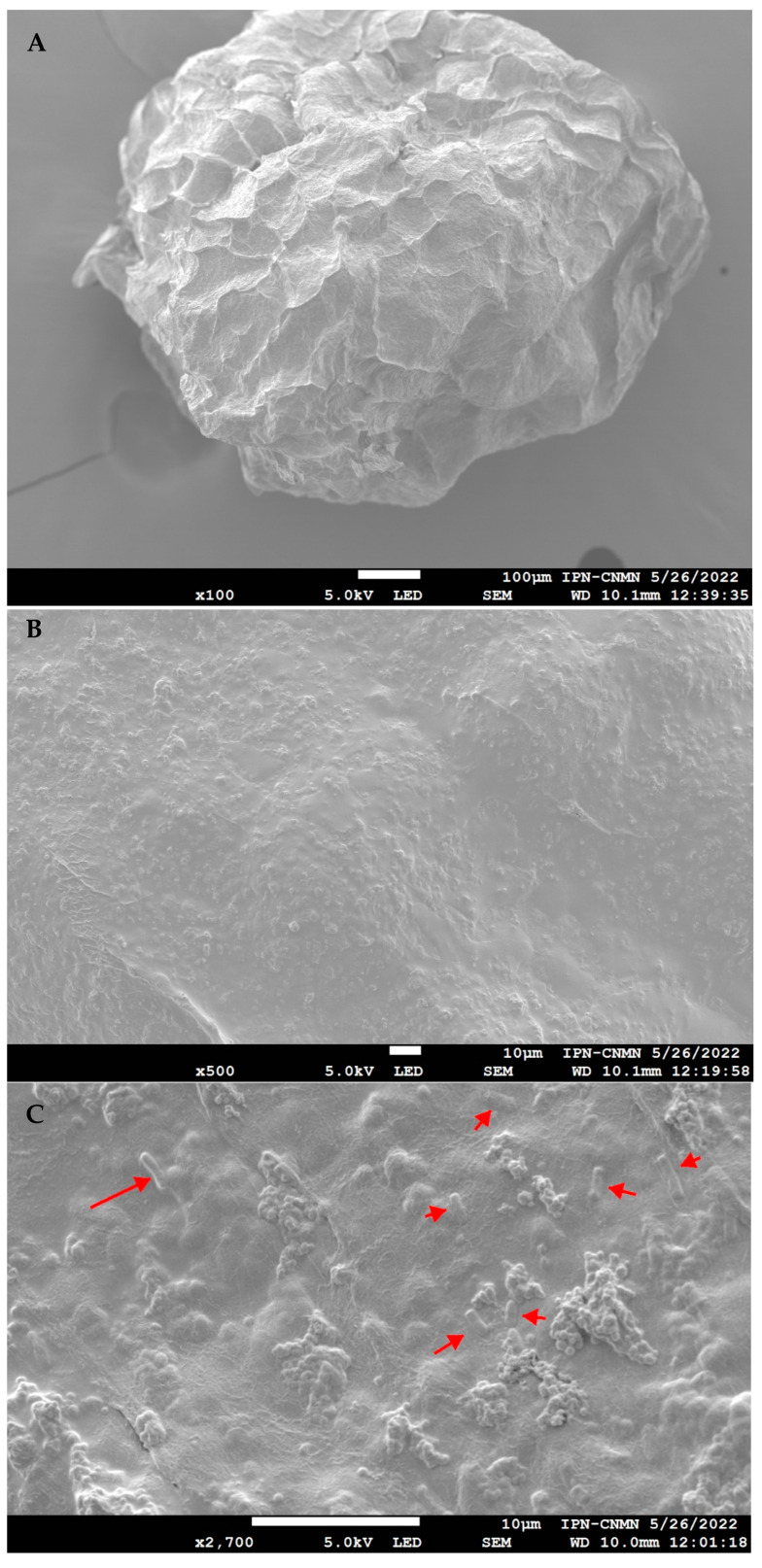
SEM Images of *L. fermentum* K73 freeze-dried SW-SA beads: (**A**) 100X, (**B**) 500X, (**C**) 2700X.

The freeze-dried beads produced by extrusion were also analyzed using scanning electron microscopy; these images are presented in Figure 3. The post-encapsulation treatment resulted in an irregular shape. However, the scanning electron microscopy images reveal a rough surface texture, which could be a beneficial characteristic of the matrix. This textural feature may facilitate the accumulation of bacteria through interactions with globular protein aggregates, enhancing the effectiveness of probiotic encapsulation [12,39]. Figures S1 and S2 show SEM images of the culture medium and optimal mixture at different magnifications.

**Figure 4:**
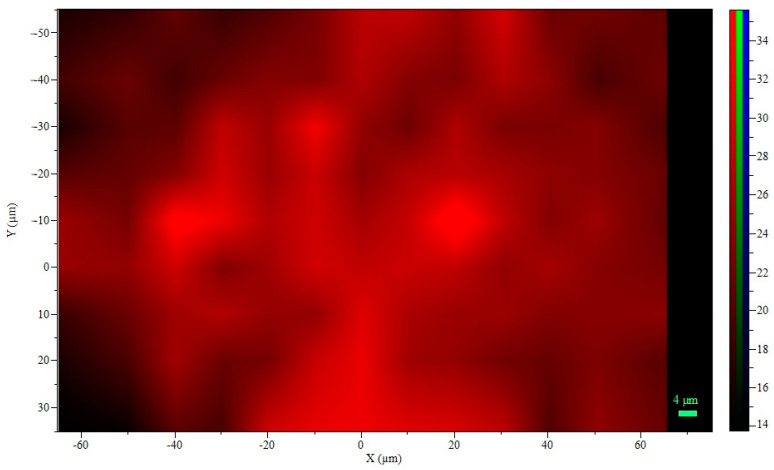
Raman mapping: 2D sodium alginate intensity.

Rojas-Muñoz et al. [20] analyzed the FTIR spectra of the bead mixture, identifying the main peaks associated with the constituent materials. Since sodium alginate is the major component in the beads, RAMAN spectroscopy mapping was conducted to visualize the spatial distribution of the polymeric component in the hydrogel microspheres used for probiotic cell encapsulation (Figure 4). The symmetric stretching band ν(COO-) at approximately 1420 cm^−1^ in the Raman spectrum was selected to specifically locate the alginate within the microspheres (see Figure S5, Sodium alginate RAMAN mapping spectra).

In general, a homogeneous intensity is observed in the mapping area (see Figure S4), with a decrease in color intensity at the corners, which may be attributed to the spherical shape of the beads, as the analysis was performed on a 2D transverse plane. To ensure stability during data acquisition, it was necessary to create a convex structure on the slide to accommodate the individual microsphere and prevent movement. This finding aligns with the study of [40], who established this technique as a non-invasive method to provide qualitative data on the spatial distribution of polymers in non-covalently crosslinked hydrogel microspheres for cellular applications.

### 3.3. Probiotic Cell Viability under INFOGEST Simulated Gastrointestinal Model

**Figure 5:**
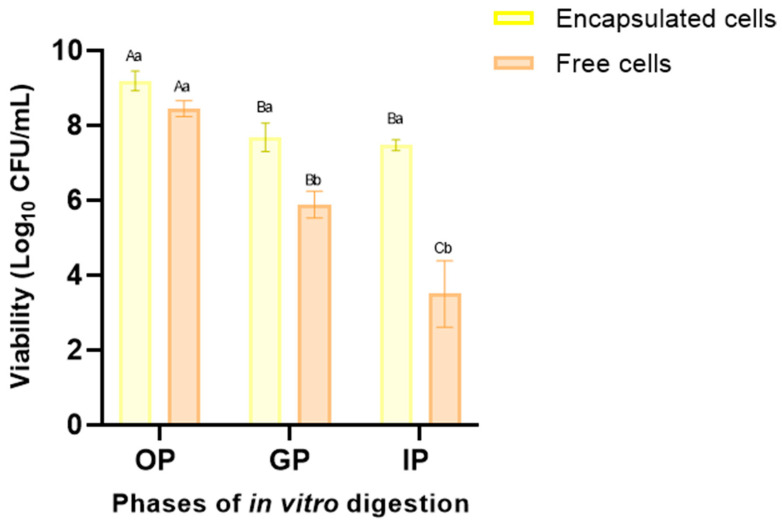
Viability of free and encapsulated *L. fermentum* K73 cells during OP (oral phase: 2 min, pH 7.0), GP (gastric phase: 120 min, pH 3.0), and IP (intestinal phase: 120 min, pH 7.0) according to the INFOGEST in vitro model. Mean value ± standard deviation of at least three independent measurements is included. (A–C) Different letters within the same treatment (encapsulated or free cells) indicate statistical significance (*p* < 0.05). (a,b) Different letters with the same in vitro digestion phase indicate statistical significance (*p* < 0.05).

Free and encapsulated *L. fermentum* K73 cells were subjected to the standardized in vitro digestion protocol developed by the INFOGEST 2.0 international network to evaluate the ability of the matrix to protect the probiotic cells during GI transit (see Figure 5). For each GI tract phase, the viability of free probiotic cells significantly decreased (*p* ≤ 0.05) compared to the encapsulated cells in SW-SA beads.

There was no significant decrease in the oral phase for either treatment. The viability of the encapsulated cells was 9.34 Log_10_ (CFU/mL), while that of the free cells was 8.91 Log_10_ (CFU/mL), compared to the initial bacteria viability of 9.6 Log_10_ (CFU/mL). This represents a reduction of 2.5% and 6.1%, respectively. After 2 h of incubation in the gastric phase (pH 3), free cells showed a significant decrease in viability (38.5% or 2.92 cycles) compared with SW-ALG encapsulated cells (18.8% or 1.57 cycles). Encapsulation with alginate and sweet whey by vibrational technology proved effective in protecting probiotic cells, as the reduction in the viability for encapsulated bacteria after digestion was significantly lower (21.0%) than that observed in free cells (63.4%) In other words 79.0% of the encapsulated cells survived, compared to 36.6% of the free cells.

Importantly, the viable count of encapsulated cells in the intestinal phase was 7.56 Log_10_ (CFU/mL), which exceeds the limit required to classify a product as probiotic (>6 Log (CFU/mL)). This result highlights the positive impact of both the bead mixture and the optimal encapsulation conditions under digestion. Considering the wall components and their established interactions, it appears that a favorable barrier is formed at the periphery of the microspheres, between the whey and alginate, which protects the microorganisms from gastrointestinal conditions and results in high encapsulation efficiency. Similar findings were reported by [15,41,42,43], who demonstrated that encapsulation by ionotropic extrusion vibrational technology enhances cell viability during simulated gastrointestinal passage.

## 4. Conclusions

In conclusion, to achieve a successful encapsulation process, the factors that influence the viability of the probiotic during each stage of the process must be considered. These factors include the selection of the culture medium, the wall materials as well as their proportions, the operating conditions of the encapsulation, and the post-encapsulation treatment. The results reported in this study showed the effect of operational conditions on the viability of *Lactobacillus fermentum* K73. The optimization of the encapsulation process resulted in high cell viability and a high encapsulation yield of the probiotic. The particles produced with the optimal mixture and conditions exhibited a suitable structure and enhanced the tolerance of the probiotics to simulated gastrointestinal conditions, thereby ensuring the requirements for functional probiotic foods.

## Figures and Tables

**Table 1:** Optimal operating conditions for BUCHI B-395 Pro Encapsulator.

Corrida	Factors			Response Variable	
	Frequency (Hz)	E. Tension (V)	Pump Rate (mL/min)	Viability (Log_10_ (CFU/mL))	EE (%)
1	4000.0	250.0	20.0	8.21	88.82
2	2428.0	250.0	11.6	7.83	86.54
3	2428.0	250.0	11.6	7.99	88.42
4	1484.8	2500.0	6.0	8.35	92.32
5	2801.4	1678.8	11.2	7.87	87.07
6	1425.9	1217.5	6.0	7.91	87.50
7	70.0	250.0	6.0	7.97	88.12
8	4000.0	2500.0	6.0	8.34	92.27
9	2801.4	1678.8	11.2	8.09	89.41
10	70.0	2500.0	20.0	7.56	83.63
11	70.0	1600.0	11.6	8.14	90.02
12	2428.0	1600.0	20.0	8.05	89.05
13	4000.0	1037.5	6.0	7.80	86.24
14	4000.0	2500.0	15.1	8.17	90.38
15	463.0	475.0	13.0	7.88	87.13
16	1484.8	2500.0	6.0	8.73	96.51
17	2428.0	1600.0	20.0	8.07	89.27
18	463.0	1375.0	18.6	8.22	90.87
19	70.0	1600.0	11.6	8.10	89.54
20	70.0	250.0	20.0	8.69	96.09

**Table 2:** Analysis of variance (ANOVA) for the optimal design.

	Viability (Log_10_ (CFU/mL))			Encapsulation Efficiency (%)		
	Sum of Squares	Degree Freedom	*p*-Value	Sum of Squares	Degree Freedom	*p*-Value
Model	1.09	9	0.025	147.56	9	0.014
AB	0.125	1	0.077	20.58	1	0.038
AC	0.001	1	0.668	2.17	1	0.457
BC	0.739	1	0.001	96.48	1	0.001
Residuals	0.322	10		36.35	10	
Lack of fit	0.215	5	0.228	22.93	5	0.285
Pure Error	0.106	5		13.42	5	
R^2^	0.813			0.802		

## Data Availability

The original contributions presented in the study are included in the article/Supplementary Material, further inquiries can be directed to the corresponding authors.

## Supplementary Materials

The following supporting information can be downloaded at: https://www.mdpi.com/article/10.3390/polym16172492/s1, Figure S1: SEM images of the L. fermentum K73 culture medium at different magnifications (A) 500X, (B) 2000X, (C) 5000X; Figure S2. SEM images of the Optimal Mixture (SW-SA: 0.39/0.61) at different magnifications (A) 20X, (B) 30X, (C) 2000X, (D) 5000 X; Figure S3. Confocal laser scanning microscopy 3D image for SW-SA bacterial beads; Figure S4. Raman two-dimensional map area; Figure S5. Sodium alginate RAMAN mapping spectra; Figure S6. RAMAN analysis of sodium alginate (SA), culture medium -mainly sweet whey (SW) and fresh SW-SA microcapsules.

## References

[1] Cueto C., Aragón S. (2012). Evaluación del potencial probiótico de bacterias ácido lácticas para reducir el colesterol in vitro Evaluation of probiotic potential of lactic acid bacteria to reduce in vitro cholesterol. Sci. Agropecu..

[2] Kim J., Muhammad N., Hak B., Yoo J.J. (2016). Probiotic delivery systems: A brief overview Probiotic delivery systems: A brief overview. J. Pharm. Investig..

[3] Misra S., Pandey P., Mishra H.N. (2021). Novel approaches for co-encapsulation of probiotic bacteria with bioactive compounds, their health benefits and functional food product development: A review. Trends Food Sci. Technol..

[4] Latif A., Shehzad A., Niazi S., Zahid A., Ashraf W., Iqbal M.W., Rehman A., Riaz T., Aadil R.M., Khan I.M. (2023). Probiotics: Mechanism of action, health benefits and their application in food industries. Front. Microbiol..

[5] Huang S., Vignolles M.L., Chen X.D., Le Loir Y., Jan G., Schuck P., Jeantet R. (2017). Spray drying of probiotics and other food-grade bacteria: A review. Trends Food Sci. Technol..

[6] Oberoi K., Tolun A., Altintas Z., Sharma S. (2021). Effect of Alginate-Microencapsulated Hydrogels on the Survival of *Lactobacillus rhamnosus* under Simulated. Foods.

[7] Razavi S., Janfaza S., Tasnim N., Gibsonbc D.L., Hoorfar M. (2021). Nanomaterial-based encapsulation for controlled gastrointestinal delivery of viable probiotic bacteria. Nanoscale Adv..

[8] Agriopoulou S., Tarapoulouzi M., Varzakas T., Jafari S.M. (2023). Application of Encapsulation Strategies for Probiotics: From Individual Loading to Co-Encapsulation. Microorganisms.

[9] Comunian T.A., Favaro-Trindade C.S. (2016). Microencapsulation using biopolymers as an alternative to produce food enhanced with phytosterols and omega-3 fatty acids: A review. Food Hydrocoll..

[10] Zabot G.L., Schaefer Rodrigues F., Polano Ody L., Vinícius Tres M., Herrera E., Palacin H., Córdova-Ramos J.S., Best I., Olivera-Montenegro L. (2022). Encapsulation of Bioactive Compounds for Food and Agricultural Applications. Polymers.

[11] Heidebach T., Först P., Kulozik U. (2012). Microencapsulation of probiotic cells for food applications. Crit. Rev. Food Sci. Nutr..

[12] Krasaekoopt W., Watcharapoka S. (2014). Effect of addition of inulin and galactooligosaccharide on the survival of microencapsulated probiotics in alginate beads coated with chitosan in simulated digestive system, yogurt and fruit juice. LWT—Food Sci. Technol..

[13] Sun Q., Yin S., He Y., Cao Y., Jiang C. (2023). Biomaterials and Encapsulation Techniques for Probiotics: Current Status and Future Prospects in Biomedical Applications. Nanomaterials.

[14] Jiménez-Martín E., Gharsallaoui A., Rojas T.A. (2015). Suitability of Using Monolayered and Multilayered Emulsions for Microencapsulation of ω-3 Fatty Acids by Spray Drying: Effect of Storage at Different Temperatures. Food Bioprocess Technol..

[15] Lee Y., Ji Y.R., Lee S., Choi M.J., Cho Y. (2019). Microencapsulation of probiotic lactobacillus acidophilus kbl409 by extrusion technology to enhance survival under simulated intestinal and freeze-drying conditions. J. Microbiol. Biotechnol..

[16] Kowalska E., Ziarno M., Ekielski A., Żelaziński T. (2022). Materials Used for the Microencapsulation of Probiotic Bacteria in the Food Industry. Molecules.

[17] Ergin F., Atamer Z., Göcer E.M.C., Demir M., Hinrichs J., Kucukcetin A. (2021). Optimization of Salmonella bacteriophage microencapsulation in alginate-caseinate formulation using vibrational nozzle technique. Food Hydrocoll..

[18] Whelehan M., Marison I.W. (2011). Microencapsulation using vibrating technology. J. Microencapsul..

[19] Kailasapathy K. (2002). Microencapsulation of Probiotic Bacteria: Technology and Potential Applications. Curr. Issues Intest. Microbiol..

[20] Rojas-Muñoz Y.V., Santagapita P.R., Quintanilla-Carvajal M.X. (2023). Probiotic Encapsulation: Bead Design Improves Bacterial Performance during In Vitro Digestion. Polymers.

[21] Aragón-Rojas S., Quintanilla-Carvajal M.X., Hernández-Sánchez H. (2018). Multifunctional Role of the Whey Culture Medium in the Spray-Drying Microencapsulation of Lactic Acid Bacteria. Food Technol. Biotechnol..

[22] Benucci I., Cerreti M., Maresca D., Mauriello G., Esti M. (2019). Yeast cells in double layer calcium alginate–chitosan microcapsules for sparkling wine production. Food Chem..

[23] Martín M.J., Lara-Villoslada F., Ruiz M.A., Morales M.E. (2015). Microencapsulation of bacteria: A review of different technologies and their impact on the probiotic effects. Innov. Food Sci. Emerg. Technol..

[24] Bevilacqua A., Campaniello D., Speranza B., Racioppo A., Altieri C., Sinigaglia M., Corbo M.R. (2020). Microencapsulation of Saccharomyces cerevisiae into Alginate Beads: A Focus on Functional Properties of Released Cells. Foods.

[25] Graff S., Hussain S., Chaumeil J., Charrueau C. (2008). Increased Intestinal Delivery of Viable Saccharomyces boulardii by Encapsulation in Microspheres. Pharm. Res..

[26] Ricaurte L., Santagapita P.R., Díaz L.E., Quintanilla-Carvajal M.X. (2020). Edible gelatin-based nanofibres loaded with oil encapsulating high-oleic palm oil emulsions. Colloids Surf. A Physicochem. Eng. Asp..

[27] Zazzali I., Rocio T., Calvo A., Manuel V., Ruíz-henestrosa P., Santagapita P.R., Perullini M. (2019). Effects of pH, extrusion tip size and storage protocol on the structural properties of Ca (II)-alginate beads. Carbohydr. Polym..

[28] Gaona-Sánchez V.A., Calderón-Domínguez G., Morales-Sánchez E., Moreno-Ruiz L.A., Terrés-Rojas E., Salgado-Cruz M.D.L.P., Escamilla-García M., Barrios-Francisco R. (2020). Physicochemical and superficial characterization of a bilayer film of zein and pectin obtained by electrospraying. Appl. Polym. Sci..

[29] Hernández-Varela J.D., Villaseñor-Altamirano S.L., Chanona-Pérez J.J., González Victoriano L., Perea Flores M.d.J., Cervantes Sodi F., Calderón Benavides H.A., Morgado Aucar P. (2022). Effect of cellulose nanoparticles from garlic waste on the structural, mechanical, thermal, and dye removal properties of chitosan/alginate aerogels. J. Polym. Res..

[30] González-Quijano G.K., Dorantes-Alvarez L., Hernández-Sánchez H., Jaramillo-Flores M.E., de Jesús Perea-Flores M., Vera-Ponce de León A., Hernández-Rodríguez C. (2014). Halotolerance and survival kinetics of lactic acid bacteria isolated from jalapeño pepper (*Capsicum annuum* L.) fermentation. J. Food Sci..

[31] Rajmohan D., Bellmer D. (2019). Characterization of Spirulina-Alginate Beads Formed Using Ionic Gelation. Int. J. Food Sci..

[32] Brodkorb A., Egger L., Alminger M., Alvito P., Assunção R., Ballance S., Bohn T., Bourlieu-Lacanal C., Boutrou R., Carrière F. (2019). INFOGEST static in vitro simulation of gastrointestinal food digestion. Nat. Protoc..

[33] Minekus M., Alminger M., Alvito P., Ballance S., Bohn T., Bourlieu C., Carriere F., Boutrou R., Corredig M., Dupont D. (2014). A standardised static in vitro digestion method suitable for food—An international consensus. Food Funct..

[34] Gañan-Calvo A.M., Riesco-Chueca P. (2006). Jetting–dripping transition of a liquid jet in a lower viscosity co-flowing immiscible liquid: The minimum flow rate in flow focusing. J. Fluid Mech..

[35] Fangmeier M., Lehn D.N., Maciel M.J., Volken de Souza C.F. (2019). Encapsulation of Bioactive Ingredients by Extrusion with Vibrating Technology: Advantages and Challenges. Food Bioprocess Technol..

[36] Olivares A., Silva P., Altamirano C. (2017). Microencapsulation of probiotics by efficient vibration technology. J. Microencapsul..

[37] Shi L., Li Z., Li D., Xu M., Chen H., Zhang Z., Tang Z. (2013). Encapsulation of probiotic *Lactobacillus bulgaricus* in alginate—Milk microspheres and evaluation of the survival in simulated gastrointestinal conditions. J. Food Eng..

[38] Nemethova V., Lacik I., Razga F. (2014). Vibration technology for microencapsulation: The restrictive role of viscosity. J. Bioprocess. Biotech..

[39] Eckert C., Agnol W.D., Dallé D., Serpa V.G., Maciel M.J., Lehn D.N., Souza C.F.V. (2018). Development of alginate-pectin microparticles with dairy whey using vibration technology: Effects of matrix composition on the protection of *Lactobacillus* spp. from adverse conditions. Food Res. Int..

[40] Kroneková Z., Pelach M., Mazancová P., Uhelská L., Treľová D., Rázga F., Némethová V., Szalai S., Chorvát D., McGarrigle J.J. (2018). Structural changes in alginate-based microspheres exposed to in vivo environment as revealed by confocal Raman microscopy. Sci. Rep..

[41] Doherty S.B., Gee V.L., Ross R.P., Stanton C., Fitzgerald G.F., Brodkorb A. (2011). Development and characterisation of whey protein micro-beads as potential matrices for probiotic protection. Food Hydrocoll..

[42] De Prisco A., Maresca D., Ongeng D., Mauriello G. (2015). Microencapsulation by vibrating technology of the probiotic strain *Lactobacillus reuteri* DSM 17938 to enhance its survival in foods and in gastrointestinal environment. LWT—Food Sci. Technol..

[43] Silva M.P., Tulini F.L., Martins E., Penning M., Fávaro-Trindade C.S., Poncelet D. (2018). Comparison of extrusion and co-extrusion encapsulation techniques to protect *Lactobacillus acidophilus* LA3 in simulated gastrointestinal fluids. LWT—Food Sci. Technol..

