# Supplementary material for: Probiotic Encapsulation: Bead Design Improves Bacterial Performance during In Vitro Digestion (Part 2: Operational Conditions of Vibrational Technology)
Source: Polymers (Basel). 2024 Aug 31;16(17):2492. doi: 10.3390/polym16172492 (PMC11397813; doi:10.3390/polym16172492)
Supplement: Supplementary file 1 [file polymers-16-02492-s001.zip › polymers-3132931-supplementary.pdf]

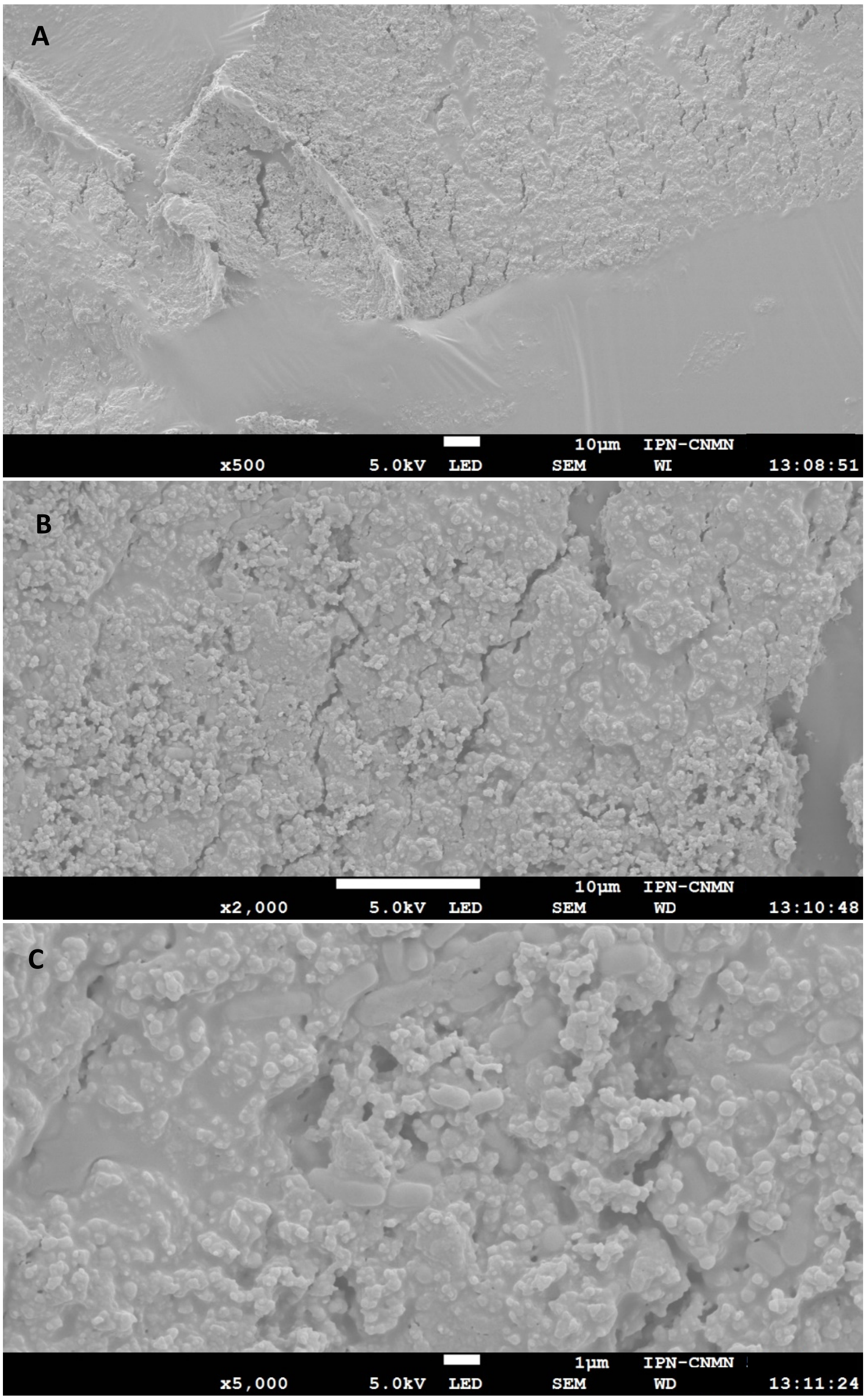

Figure S1 SEM images of the *L. fermentum* K73 culture medium at different magnifications: (A) 500X, (B) 2000X, (C) 5000X

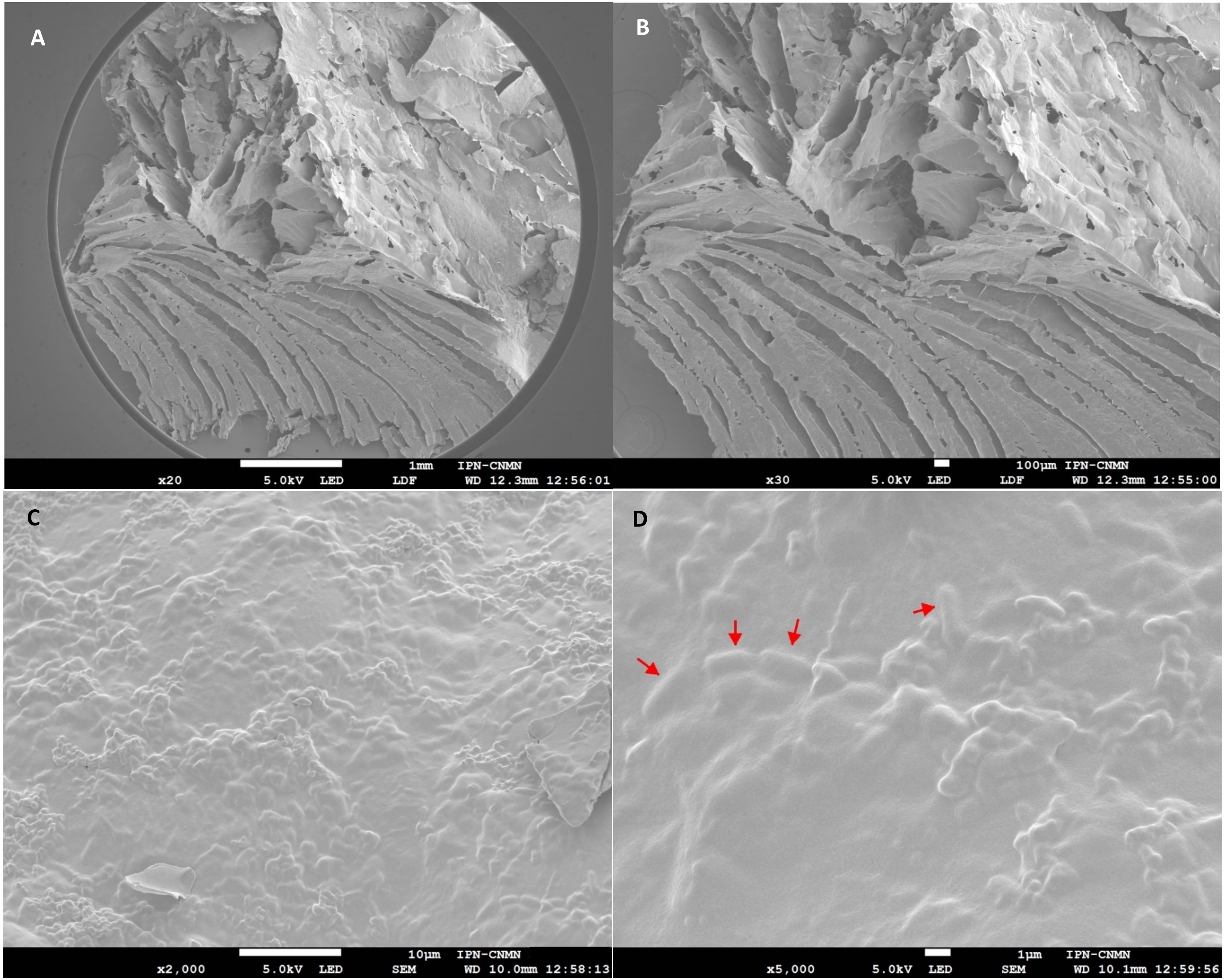

Figure S2 SEM images of the optimal mixture (SW-SA: 0.39/0.61) at different magnifications: (A) 20X, (B) 30X, (C) 2000X, (D) 5000 X

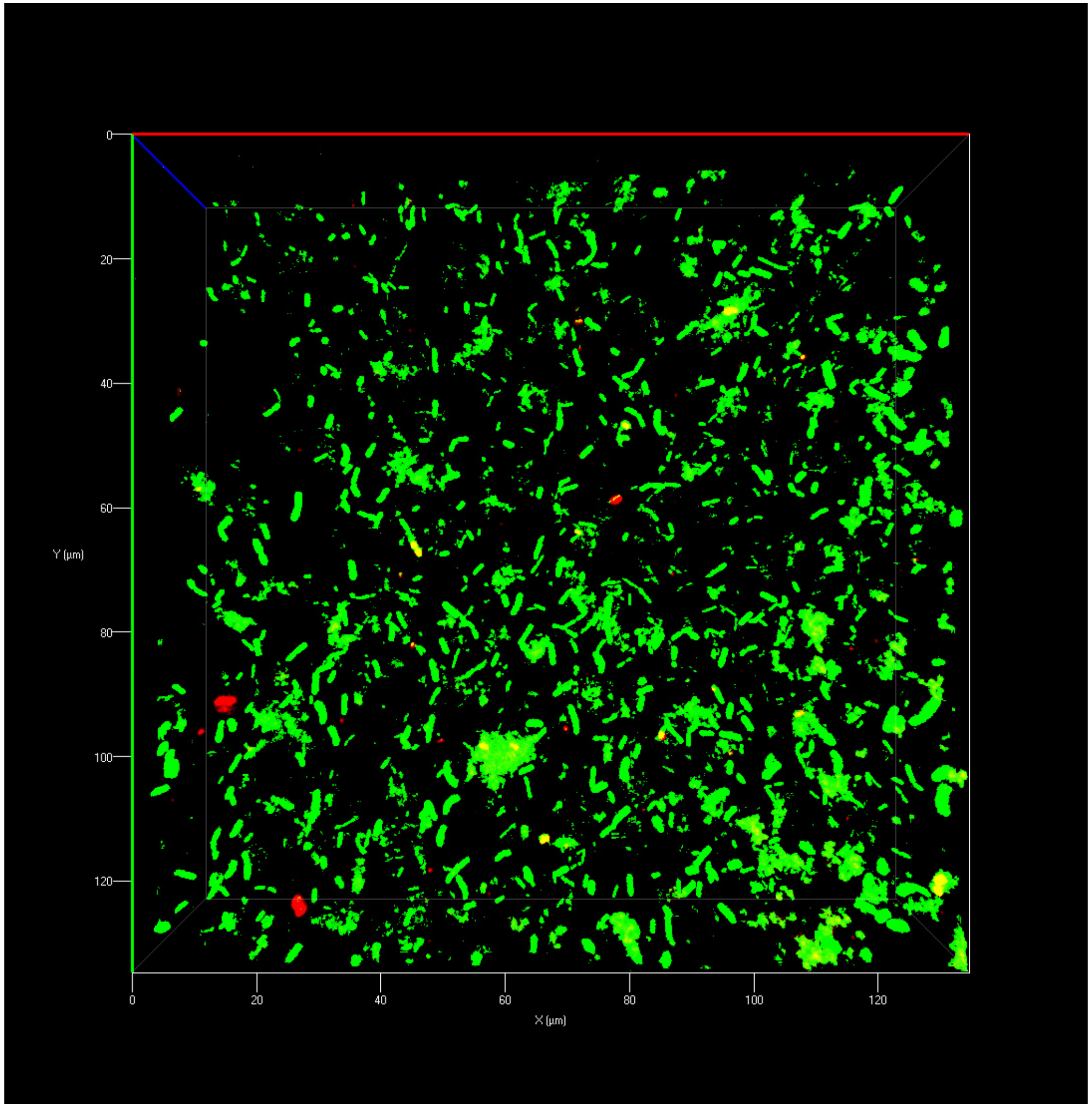

Figure S3 Confocal laser scanning microscopy 3D image for SW-SA bacterial beads

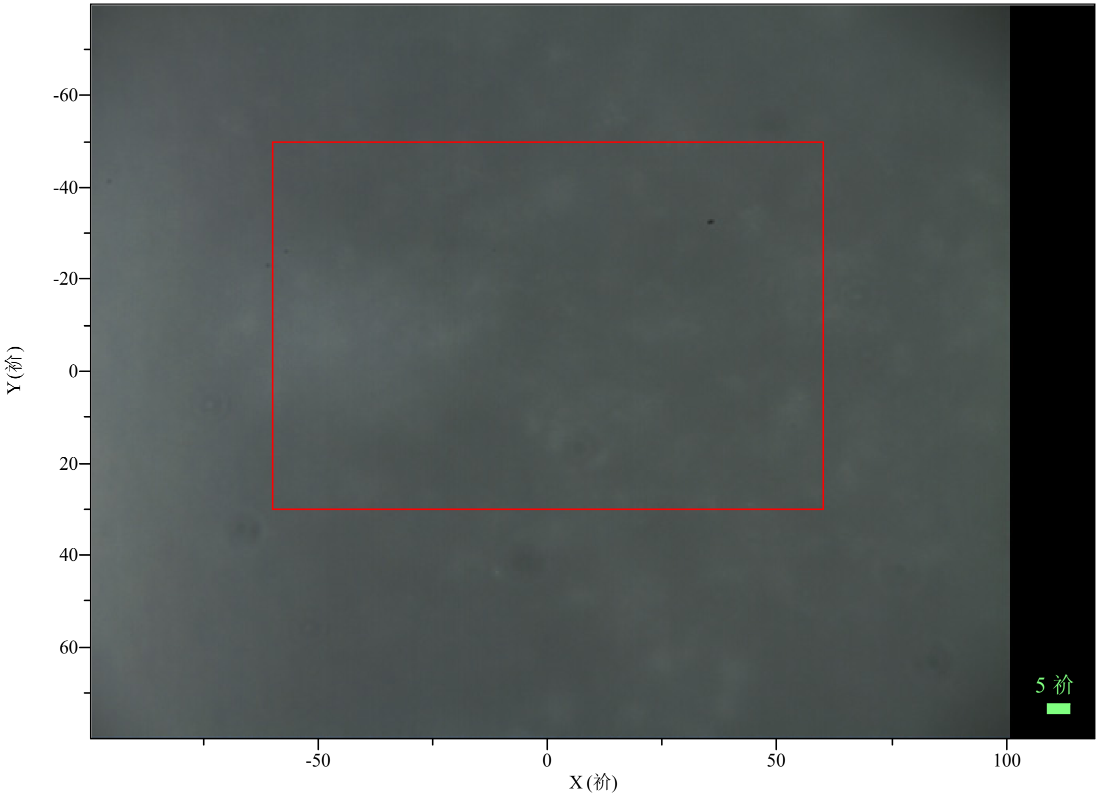

Figure S4 Raman two-dimensional map area

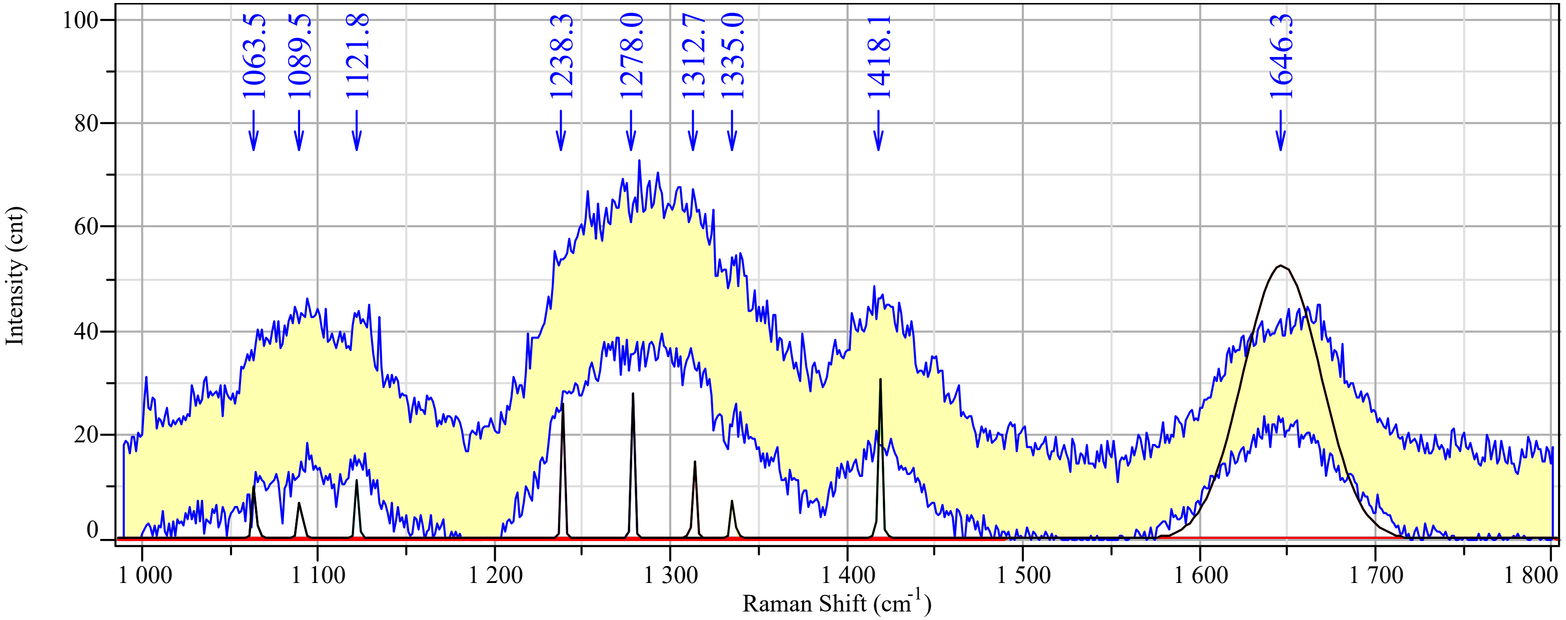

Figure S5 Sodium alginate RAMAN mapping spectra

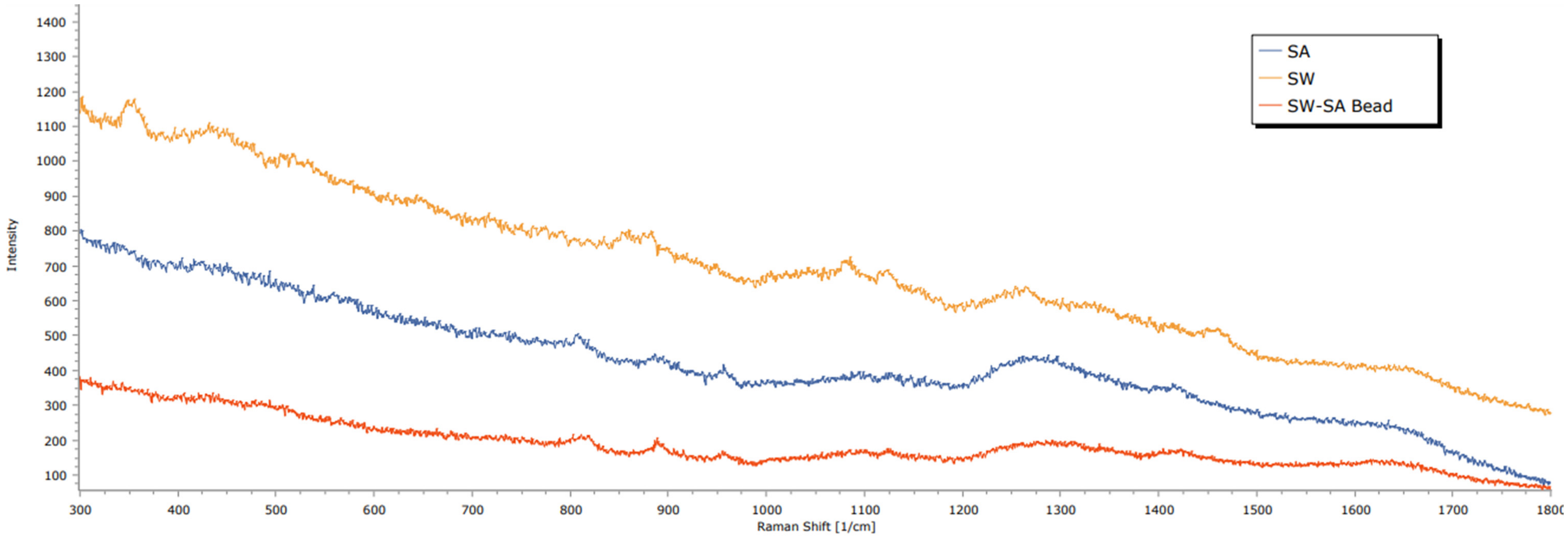

Figure S6 RAMAN analysis of sodium alginate (SA), culture medium, mainly sweet whey (SW), and fresh SW-SA microcapsules
